# EBV‐associated CNS infection in an immunocompetent adult: A case report and literature review

**DOI:** 10.1002/ccr3.8568

**Published:** 2024-03-04

**Authors:** Gwyn Srifuengfung, Pichatorn Suppakitjanusant, Nattanicha Chaisrimaneepan

**Affiliations:** ^1^ Department of Neurology Texas Tech University Health Sciences Center Lubbock Texas USA; ^2^ Department of Medicine Texas Tech University Health Sciences Center Lubbock Texas USA

**Keywords:** altered mental status, aseptic meningitis, CNS infection, CSF PCR, Epstein–Barr virus, viral encephalitis

## Abstract

EBV infections rarely cause CNS involvement. For young adult patients with suspected CNS infection, bacterial and other common viral infections should be excluded first and treated empirically until proven otherwise. Challenges in diagnosing EBV‐associated CNS infection, emphasizing the role of CSF PCR in confirming the diagnosis.

## 1 INTRODUCTION

EBV infection is commonly characterized by fever, malaise, sore throat, upper respiratory symptoms, headache, and lymphadenopathy. CNS involvement is uncommon, occurring in 0.5%–7.5% of all EBV infections,^1^ and can present with a wide spectrum of symptoms, including encephalitis, meningitis, myelitis, cranial neuropathy, mononeuritis multiplex, brachial plexopathy, acute psychosis, Guillain Barré syndrome, to acute cerebellar ataxia or seizures.^1^, ^2^, ^3^ These neurological symptoms usually manifest 1–3 weeks after the onset of respiratory symptoms^1^, ^4^ but could also manifest much later, as in the case of our patient. Interestingly, EBV‐related CNS infection tends to occur in younger adults, with an average age of 36, as reported in Japan.^4^ We report a case of EBV‐associated CNS infection in an immunocompetent adult.

## 2 CASE PRESENTATION

An 18‐year‐old Caucasian male with a past medical history of infectious mononucleosis 8 weeks ago presented with altered mental status. His friends found him lying on the floor with a fever, groaning, combative, and not able to be calmed with verbal measures. He was alert and oriented as recently as the day prior. He had no other significant past medical history, history of drug or alcohol abuse, and no prior trauma. Emergency medical services (EMS) brought him to an outside facility, where he was sedated and intubated to protect his airway with a GCS of 7 and transferred to our facility. Physical examination demonstrated a fever of 39.16°C (102.5°F), no lymphadenopathy, mild hepatosplenomegaly, and nuchal rigidity. Initial neurological examination under sedation was otherwise non‐focal.

## 3 METHODS

The patient had elevated leukocytosis with no left shift. Atypical lymphocytes were detected. A mononucleosis screen (heterophile antibodies) test was positive. The glucose level was mildly elevated. Serum salicylate, alcohol, and acetaminophen levels were negative. His serum creatinine, creatinine kinase, and aldolase levels were elevated (Table 1). The urine drug screen test, which included amphetamine, barbiturate, benzodiazepine, cannabinoid, cocaine, and opiate, was negative. A computed tomography (CT) scan without contrast of the head showed no intracranial abnormalities. He was treated empirically with piperacillin‐tazobactam and intravenous fluid resuscitation before being transferred to the intensive care unit (ICU).

**TABLE 1:** Basic bloodwork: complete blood count (CBC) and serum chemistries.

Investigation	Normal range	Day 0	Day 3	Day 7
Blood count				
WBC count	4.5–11 K/μL	17.5 K/μL	14.35 K/μL	8.57 K/μL
Neutrophils	37%–80%	46%	91.90%	‐
Lymphocytes	10%–50%	39%	4.50%	‐
Atypical lymphocytes	0%	6%	‐	‐
Monocytes	2%–9%	8%	2.90%	‐
Eosinophils	0%–4%	1%	‐	‐
RBC count	4.7–6.1 M/μL	5.26 M/μL	4.75 M/μL	5.02 M/μL
Hemoglobin	14.0–18.0 g/dL	15.7 g/dL	14.5 g/dL	14.9 g/dL
Hematocrit	42.0–52.0%	46.50%	41.40%	40.90%
MCV	80–94 fL	86.70%	88.30%	81.50%
Platelets	140–450 K/μL	359 K/μL	207 K/μL	238 K/μL
Serum chemistries				
Glucose	65–115 mg/dL	138 mg/dL	133 mg/dL	115 mg/dL
BUN	8–21 mg/dL	18 mg/dL	13 mg/dL	11 mg/dL
Creatinine	0.8–1.5 mg/dL	1.5 mg/dL	0.9 mg/dL	0.9 mg/dL
AST (SGOT)	17–59 units/L	35 units/L	211 units/L	103 units/L
ALT (SGPT)	21–72 units/L	34 units/L	99 units/L	122 units/L
Alkaline phosphatase	38–126 units/L	68 units/L	62 units/L	52 units/L
Creatinine kinase	26–308 IU/L	2953 IU/L	12,080 IU/L	1911 IU/L
Aldolase	8.1 units/mL	‐	138.8 units/mL	‐

On the first day of arrival to the ICU, the patient remained sedated due to severe agitation. Piperacillin‐tazobactam was switched to Ceftriaxone and Vancomycin, and Dexamethasone was added for suspected bacterial meningitis. Serum HIV, HSV‐1, HSV‐2, CMV, Syphilis screen, and coccidiosis were negative. The patient tested positive for serum Epstein Barr Virus (EBV) Viral Capsid Antigen (VCA) IgG, EBV VCA IgM, and EBV Nuclear Antigen (EBNA) IgG. His serum creatinine kinase remained elevated (Table 1). Urinalysis was positive for blood and red blood cells (RBC). He received aggressive fluid hydration for rhabdomyolysis due to combative behavior and sedation.

**FIGURE 1:**
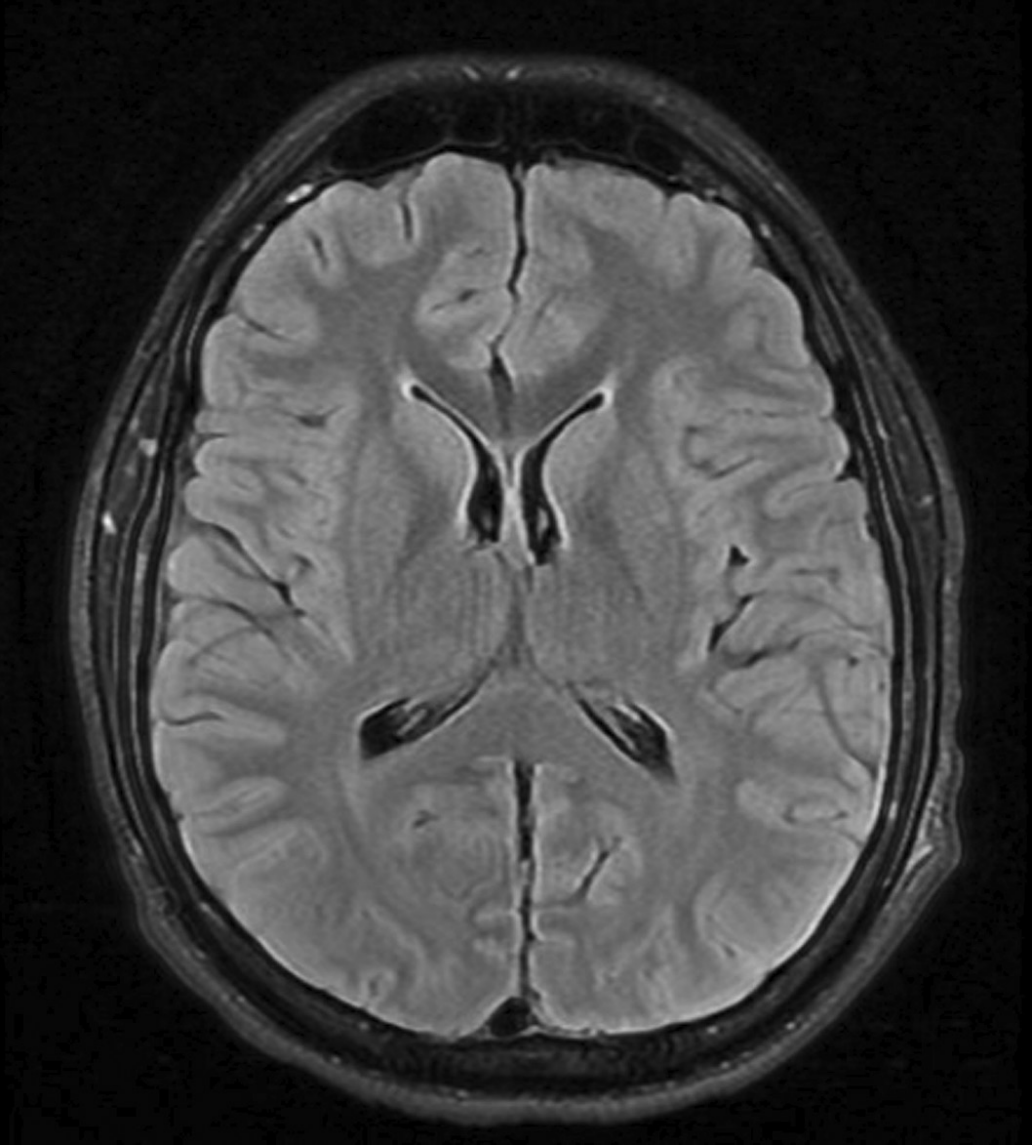
MRI head T2 FLAIR on axial view shows normal parenchyma and ventricles with no extra‐axial fluid collection, mass lesion or mass effect.

**FIGURE 2:**
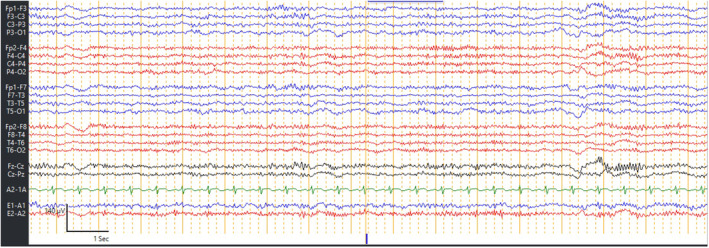
Routine EEG showing generalized slowing with absent PDR.

The magnetic resonance imaging (MRI) of the head was normal, with no restricted diffusion in the diffusion‐weighted image (DWI), and the apparent diffusion coefficient (ADC) map (Figure 1). The electroencephalography (EEG) only showed continued generalized slowed activity and absent post‐dominant rhythm consistent with encephalopathy (Figure 2).

A lumbar puncture was performed to obtain cerebrospinal fluid (CSF) for analysis. The opening pressure was elevated, and the CSF was clear. CSF analysis demonstrated increased WBC with lymphocyte predominance, increased protein, and mildly increased glucose, which was consistent with aseptic meningitis (Table 2).

**TABLE 2:** Lumbar puncture opening pressure and CSF analysis.

Investigation	Normal range	Results
Opening pressure	6–25 cmH_2_O	26 cmH_2_O
CSF analysis		
WBC	0–5	42
Neutrophils	0%–6%	12%
Lymphocytes	40%–80%	85%
RBC	‐	98
Protein	12–45 mg/dL	90 mg/dL
Sugar	40–70 mg/dL	72 mg/dL
LDH	‐	26 units/L
EBV DNA quantitative	Not detected	<200 detected copies/mL
	Not detected	<2.3 logio DNA copies/mL

Intravenous acyclovir was added to cover for possible herpes meningoencephalitis. The CSF panel came back later, and EBV DNA was detected. The CSF viral panel tested for Enterovirus, Echovirus, HSV‐1, HSV‐2, Coxsackie virus, West Nile Virus, Lymphocytic choriomeningitis virus, VZV, mumps, and measles virus was negative. The bacterial meningitis panel, which included *Haemophilus influenzae* type B, *Neisseria meningitides*, *Escherichia coli*, *Streptococcus pneumoniae*, and Group B Streptococcus was unremarkable. Coccidioides Ab immunodiffusion from CSF was also negative. The acid‐fast bacilli (AFB) stain and mycobacterium culture were also negative. He was diagnosed with viral meningoencephalitis due to EBV infection.

## 4 RESULTS

The patient's mental status started to improve over the next 24 h; he became more cooperative, started responding to commands, and was successfully extubated. The neurological examination after extubating which included cranial nerves, muscle strength and sensation, and tendon reflexes were all unremarkable, although he still has neck stiffness and limited hip flexion due to muscle pain. The patient remained slightly confused and exhibited some degree of unusual behavior and language use noticeable by his parents. He complained of some muscle aches and headaches. The decision to discontinue antibiotics, acyclovir, and dexamethasone was made after 4 complete days of treatment as the etiology of his illness was likely from EBV infection.

His clinical and mental status gradually improved with supportive and symptomatic treatment. By the fourth day of admission, his mental status was back to normal baseline. He was able to walk around with minimal muscle aches and a mild posterior headache. The patient continued to exhibit no neurological deficit. Eventually, he was discharged home by the seventh day.

## 5 DISCUSSION

The mechanism underlying EBV‐associated CNS infection is not well established. Current evidence suggests that EBV gains access to the CNS by utilizing infected lymphocytes to traverse the blood–brain barrier. Postmortem studies in specific cases have identified infiltrated lymphocytes containing EBV DNA in meninges and perivascular areas, with their absence in neurons indicating inflammatory brain damage resulting from an immune response rather than direct viral invasion, in contrast to herpes simplex virus (HSV) infection.^5^, ^6^, ^7^, ^8^, ^9^ An in vitro study provides an intriguing alternative perspective, demonstrating EBV's capacity to infect various neural cells, generating progeny virus through lytic replication and causing host cell destruction, potentially infecting other neurons and mononuclear cells.^10^ In a rat encephalitis model, anti‐neuronal antibodies have been detected.^11^ Despite these controlled laboratory findings, demonstrating this phenomenon in vivo remains inconclusive with current knowledge.

EBV‐associated CNS infection can occur in 1–3 weeks following an acute infection,^12^ but can also occur in the absence of acute infection, potentially attributed to reactivation. There is currently no established diagnostic criteria of EBV‐associated CNS infection. While a possible diagnosis has been suggested based on positive serologic findings alongside compatible neurological symptoms,^13^ the rarity of this condition dictates the exclusion of more common viruses in suspected cases among young immunocompetent patients.^8^ Further support for diagnosis may be provided by a positive cerebrospinal fluid (CSF) polymerase chain reaction (PCR) for EBV DNA,^6^, ^13^ yet the specificity and sensitivity of this test remain undetermined. In a review of 23 cases diagnosed with EBV‐associated CNS infection, 20 tested positive on the CSF PCR.^11^ However, even when CSF PCR for EBV DNA is positive, co‐infection is prevalent, found in 22% of patients with compatible symptoms. In such cases, it has been suggested that, instead of being the primary pathogen, EBV may be reactivated secondary to inflammatory responses from other pathogens, or even contamination from EBV‐infected lymphocytes.^9^ While direct isolation of the virus in brain biopsy or culture from the CSF could support the diagnosis,^6^ such approaches are less practical in clinical practice.

In our patient, the EBV DNA in CSF is measured at less than 200 copies/mL,^3^, ^4^ which falls within the previously reported range of 51–216,000 copies/mL.^9^, ^14^ The absence of other pathogens on PCR and bacterial panels makes contamination unlikely. In cases involving potential co‐infections with other pathogens, we concur that utilizing reverse transcription‐polymerase chain reaction (RT‐PCR) for messenger RNA (mRNA) of the lytic cycle gene BZLF in the CSF may be the optimal approach to establishing the pathogenic role of EBV.^9^ The BZLF gene, responsible for encoding a transcription regulator protein, is expressed by EBV only during the transition from latent to lytic infection.

Our case also demonstrated the challenging nature of determining the chronicity of an EBV‐related CNS infection. For an acute infection, a positive serum EBV‐specific IgM and IgG to the viral capsid antigen (VCA IgG, IgM), a positive IgG to the early antigen (EA IgG), and a negative IgG to nuclear antigen (EBNA IgG) is suggestive. If follow‐up serology were obtained, significant changes in EBV‐specific antibodies such as a rise in IgG level, the disappearance of VCA‐IgM and EA IgG, and the appearance of EBNA IgG further support an acute infection.^13^ The positive EBNA IgG in the context of positive VCA IgM and VCA IgG in our patient may initially appear perplexing. VCA IgM is a marker of acute infection. At the same time, EBNA IgG is usually expressed only during latent infection, beginning from 6 weeks after the first infection, as EBNA are proteins responsible for maintaining an episomal state of EBV DNA as well as immortalizing B‐cells in which EBV persists during latent infection. However, this can be reconciled by the fact that VCA IgM can be presented up to 3 months following acute infection. Our patient's reported symptoms of infectious mononucleosis 8 weeks prior and mild hepatosplenomegaly on the exam are consistent with that. Another possibility is a reactivation infection. In either case, the timing of the infection is not likely to be clinically significant, and we suggest serial follow‐up serology in case chronicity needs to be elucidated.

Neuroimaging is normal in most of the patients with EBV‐associated CNS infection, as in our patient. However, it is essential to perform an MRI to rule out other causes of CNS infection such as HSV encephalitis or acute disseminated encephalomyelitis (ADEM), which typically presents with lesions mostly in the deep and subcortical white matter.^10^ The MRI findings in EBV‐associated CNS infection can vary widely in hyperintensity in the basal ganglia, thalamus, cerebral cortex, brainstem, optic nerves, splenium, and corpus callosum.^15^, ^16^ These manifestations may extend to brainstem hemorrhage,^5^ meningeal enhancement, and multi‐level spinal cord involvement.^13^ While most MRI abnormalities are observed in the FLAIR and T2 sequences, restricted diffusion on the DWI sequence can also be presented. Additionally, heterogeneous signals on ADC sequences can be observed, ranging from hypo‐intensity to hyperintensity.^17^ However, none of these findings is specific to EBV infection.

There is no standard treatment guideline for EBV‐associated CNS infection. There have been reports of ganciclovir or acyclovir treatment with or without corticosteroids. This is due to ganciclovir's ability to suppress replication in DNA viruses and good in‐vitro activity against EBV virus.^16^ However, the efficacy remains unclear.^9^, ^18^ Suppose the main pathogenetic cause of EBV‐associated CNS infection is the result of the inflammatory response by the body's immune system rather than direct viral invasion. In that case, systemic corticosteroids are more likely to be efficacious. While the exact pathogenesis of EBV‐associated CNS infection is not yet entirely clear, we believe it is reasonable, in the absence of contraindications, to initiate treatment with both antivirals and systemic corticosteroids. Our patient received both systemic corticosteroid and intravenous acyclovir empirically and they were discontinued once the patient showed continuous improvement, and we thus believe the duration of this treatment could be guided by the patient's clinical response.

## 6 CONCLUSIONS

EBV infection involves a very heterogeneous constellation of symptoms. Isolated, EBV‐associated CNS infection should be considered in young adults with altered mental status when other more common CNS viral infections have been ruled out. A positive CSF PCR for EBV DNA strongly supports the diagnosis in the absence of other pathogens in the PCR. MRI findings typical of other conditions, such as ADEM, also must be ruled out. Due to the unclear pathogenesis of EBV‐associated CNS infection, we suggest the combination of antivirals and systemic corticosteroids if not contraindicated.

## AUTHOR CONTRIBUTIONS

**Gwyn Srifuengfung:** Writing – original draft; writing – review and editing. **Pichatorn Suppakitjanusant:** Conceptualization; writing – review and editing. **Nattanicha Chaisrimaneepan:** Resources; writing – original draft; writing – review and editing.

## FUNDING INFORMATION

The authors did not receive financial support for the research, authorship, and/or publication of this article.

## CONFLICT OF INTEREST STATEMENT

There are no conflicts of interest to declare.

## CONSENT

Verbal and written consent was obtained from the patient to publish this case.

## Data Availability

All data underlying the results are available as part of the article and no additional source data are required.
